# The SGLT2 Inhibitor Dapagliflozin Disrupts the Cell Cycle at High Concentrations Without Altering Glycosphingolipid (De Novo)Biosynthesis

**DOI:** 10.3390/ijms26199811

**Published:** 2025-10-09

**Authors:** Richard Jennemann, Roger Sandhoff

**Affiliations:** Research Group Lipid Pathobiochemistry, German Cancer Research Center, Im Neuenheimer Feld 581, 69120 Heidelberg, Germany; r.sandhoff@dkfz.de

**Keywords:** glucosylceramide synthase (GCS), glycosphingolipids, dapagliflozin, miglustat, eliglustat, Genz-123346, GCS-inhibitors

## Abstract

Modern computational screening methods are valuable tools for repurposing approved drugs for novel therapeutic applications. They provide initial insights into alternative uses and may significantly shorten the lengthy process of drug development and regulatory approval. Treatment options for glycosphingolipidoses, lysosomal storage diseases involving glycosphingolipids (GSLs), are currently limited to a few drugs that inhibit de novo GSL biosynthesis, such as eliglustat and miglustat (Zavesca^®^). In the search for alternative drugs, dapagliflozin emerged as a promising candidate for off-target therapy. In the present study, we investigated whether dapagliflozin can indeed inhibit GSL synthesis, as predicted by previous computational analyses, and compared its effects with those of the glycosphingolipid synthesis inhibitor, the eliglustat analog Genz-123346, in murine 3T3 and Hepa 1-6 cell lines. While Genz-123346 significantly inhibited glycosphingolipid biosynthesis at concentrations as low as 1 µM, dapagliflozin, even up to 50 µM, had no effect on biosynthesis or de novo biosynthesis in either cell line. These results indicate that dapagliflozin, although assessing effects on the cell cycle, including proliferation at high concentrations, is not a suitable candidate for treating glycosphingolipid storage diseases by substrate reduction.

## 1. Introduction

Dapagliflozin is a selective, potent, and reversible inhibitor of the sodium-glucose co-transporter 2 (SGLT2) [1]. It is widely used in the treatment of type 2 diabetes mellitus [1,2,3] and has also been reported to exert beneficial effects on cardiac function [4,5]. More recently, computational analyses have suggested dapagliflozin as a potential candidate for targeting glucosylceramide synthase (GCS) in glycosphingolipid (GSL) storage disorders [6]. However, no direct in vitro evidence of its inhibitory activity towards GCS was provided. The primary aim of our investigations was therefore to assess whether dapagliflozin indeed has the potential to inhibit GSL synthesis.

**Figure 1:**
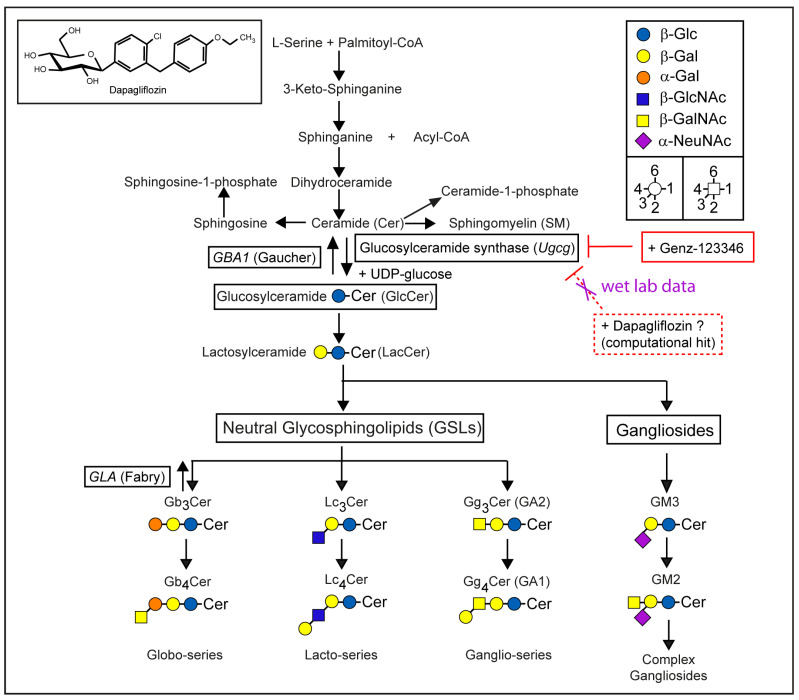
Schematic representation of the biosynthesis and degradation of glycosphingolipids (GSLs). The synthesis of glucosylceramide (GlcCer) from ceramide and UDP-glucose, catalyzed by glucosylceramide synthase (GCS), is the fundamental step in GSL biosynthesis. Deficiencies in GSL-degrading enzymes, such as glucocerebrosidase (*GBA1*) or α-galactosidase A (*GLA*), lead to the accumulation of GlcCer (Gaucher disease) or globosides (Fabry disease), respectively. While the GCS inhibitor Genz-123346 effectively blocked glycosphingolipid (GSL) synthesis, dapagliflozin showed no such effect.

GCS represents the key enzyme in the GSL biosynthesis pathway, encoded by the gene *UGCG*. GSLs are essential membrane components involved in various biological processes, including cell differentiation, adhesion, and intracellular transport [7]. GSL biosynthesis begins in the endoplasmic reticulum, where the enzyme serine-palmitoyltransferase catalyzes the condensation of serine and palmitoyl-CoA to produce a sphingoid base (Figure 1) [8]. The reduced sphingoid base is further acylated by ceramide synthases, generating ceramides [9]. Ceramides are transported, either in vesicles or by lipid transport proteins, to the Golgi apparatus, where GCS catalyzes the formation of glucosylceramide (GlcCer) on the cytosolic side of the membrane (Figure 1) [10,11]. GlcCer serves as the precursor for more complex neutral GSLs and gangliosides, through elongation by specific glycosyltransferases [8].

The impaired lysosomal degradation of GSLs, due to absent or reduced glycosidase activity, leads to their intracellular accumulation and is associated with lysosomal storage disorders such as Gaucher disease and Fabry disease. Gaucher disease results from mutations in the *GBA1* gene, leading to deficient activity of acid β-glucocerebrosidase and subsequent accumulation of glucosylceramide, which severely affects the spleen, liver, and bone marrow. Fabry disease, on the other hand, is caused by mutations in the *GLA* gene encoding α-galactosidase A, resulting in the accumulation of globotriaosylceramide in various tissues and organs. Both diseases share similar systemic symptoms. The degradation of GSLs is mediated by acid hydrolases in lysosomal compartments [12]. The dysfunction of these lysosomes, often due to inherited autosomal recessive defects in lipid-degrading enzymes, leads to lipid accumulation and can result in life-threatening consequences [13]. Treatment strategies for lysosomal storage disorders include enzyme replacement therapy (ERT), substrate reduction therapy (SRT), or combinations of both [13]. One such SRT approach involves the use of miglustat (N-butyldeoxynojirimycin), an iminosugar that inhibits GSL biosynthesis. Miglustat is approved for the treatment of Gaucher disease type 1 [14] and has also been evaluated in combination with ERT [15]. While it may benefit systemic symptoms, miglustat appears to have limited efficacy for neurological manifestations, such as those observed in type 3 Gaucher disease [16]. In the search for novel and potent GCS inhibitors capable of crossing the blood–brain barrier, computational analysis identified dapagliflozin (Figure 1) as the most promising candidate [6].

In this study, we investigated the ability of dapagliflozin to inhibit GSL synthesis in 3T3 and Hepa 1-6 cells. Our results show that dapagliflozin does not affect GSL levels. These findings indicate that dapagliflozin is unlikely to constitute a viable therapeutic option for glycosphingolipid storage disorders.

## 2. Results

### 2.1. Dapagliflozin Induces Cell Cycle Arrest in 3T3 and Hepa 1-6 Cells at High Concentrations

**Figure 2:**
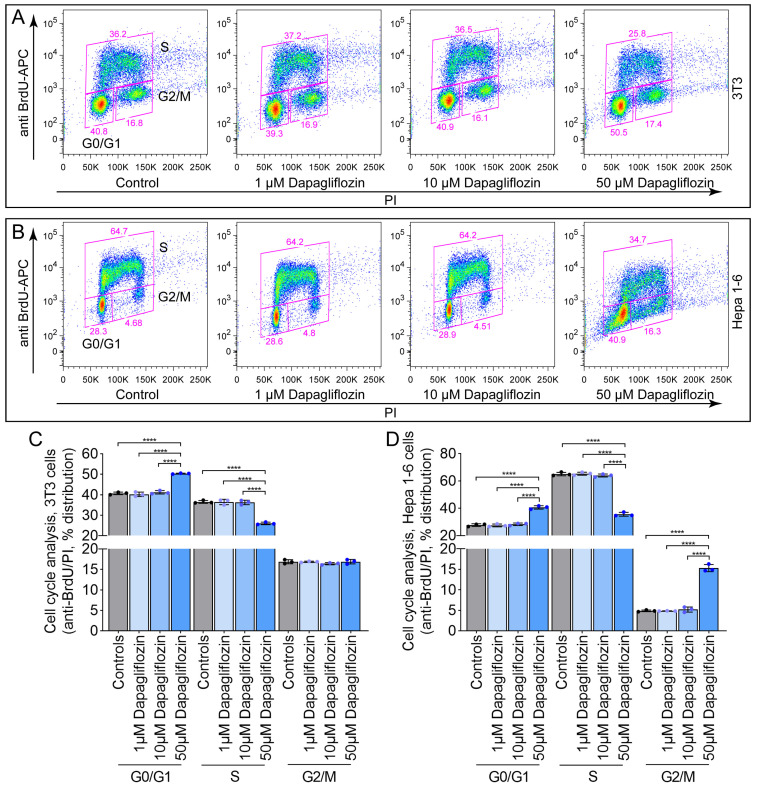
Dapagliflozin inhibits the cell cycle of 3T3 and Hepa 1-6 cells only at high concentrations. (**A**–**D**) Cell cycle analysis by 5-bromo-2′-deoxyuridine (BrdU)/PI staining. 3T3 cells (**A**) FACS images, (**C**) quantification) and Hepa 1-6 cells (**B**) FACS images, (**D**) quantification). Increasing cell density in the FACS images is shown by blue-, green-, yellow-, and red colors. Data represent one of two independent experiments; *n* = 3 per condition. Bar graphs show mean ± SD. Statistical significance was determined by one-way ANOVA: ****, *p* < 0.0001.

To examine the impact of dapagliflozin on the cell cycle, 3T3 and Hepa 1-6 cells were treated with 1 µM, 10 µM, or 50 µM dapagliflozin for six days, followed by a one-hour pulse with 5-bromo-2′-deoxyuridine (BrdU). Treatment with 1 µM or 10 µM had no significant effect on S-phase entry in either 3T3 (Figure 2A,C) or Hepa 1-6 cells (Figure 2B,D). In contrast, 50 µM dapagliflozin led to a marked increase in the proportion of cells in the G0/G1 phase and a corresponding reduction in S-phase entry (Figure 2C,D), indicating cell cycle arrest at higher concentrations.

### 2.2. Dapagliflozin Does Not Induce Pronounced Apoptosis or Necrosis in 3T3 and Hepa 1-6 Cells

**Figure 3:**
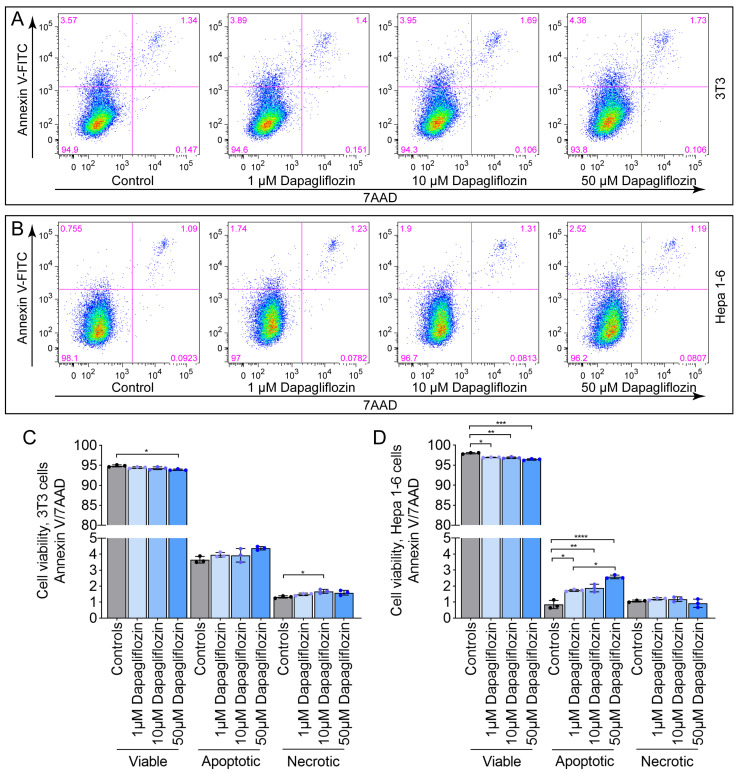
Dapagliflozin has no significant effect on apoptosis or necrosis in 3T3 and Hepa 1-6 cells. (**A**–**D**) Annexin V/7-AAD flow cytometric analysis following treatment with various concentrations of dapagliflozin. (**A**) Representative FACS plots for 3T3 cells; (**B**) representative FACS plots for Hepa 1-6 cells. (**C**) Quantification for 3T3; (**D**) quantification for Hepa 1-6. Increasing cell density in the FACS images is shown by blue-, green-, yellow-, and red colors. Data represent one of two independent experiments; *n* = 3 per condition. Bar graphs show mean ± SD. One-way ANOVA: *, *p* ≤ 0.05; **, *p* < 0.01; ***, *p* < 0.001; ****, *p* < 0.0001.

Having established that high-dose dapagliflozin induces cell cycle arrest, we next investigated whether it also triggers apoptosis or necrosis. 3T3 (Figure 3A,C) and Hepa 1-6 (Figure 3B,D) cells were treated with 1 µM, 10 µM, or 50 µM dapagliflozin for six days and subsequently analyzed by annexin V/7-AAD staining. In 3T3 cells, none of the tested concentrations led to a notable increase in apoptotic or necrotic cell populations (Figure 3C). In contrast, treatment of Hepa 1-6 cells resulted in a slight increase, particularly in apoptotic populations (Figure 3D), suggesting that dapagliflozin exerts rather a minimal effect on cell death.

### 2.3. Genz-123346 Is More Potent than Dapagliflozin in Inhibiting Cell Proliferation

**Figure 4:**
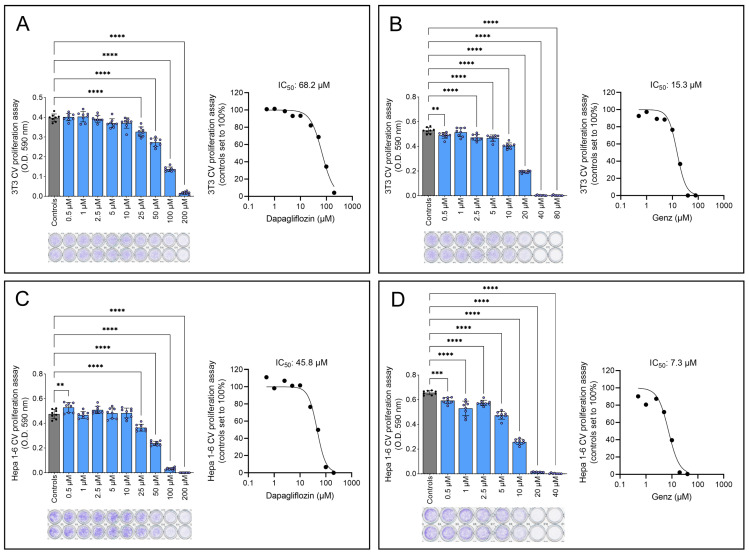
IC_50_ determination for dapagliflozin and Genz-123346 in 3T3 and Hepa 1-6 cell proliferation assays. (**A**,**B**) Effects of dapagliflozin (**A**) and the GCS inhibitor Genz-123346 (**B**) on 3T3 proliferation. (**C**,**D**) Effects of dapagliflozin (**C**) and Genz-123346 (**D**) on Hepa 1-6 proliferation. Cells were seeded in 96-well plates, allowed to adhere overnight, treated with inhibitors for 48 h, fixed with paraformaldehyde, and stained with crystal violet (CV). Shown is one representative of two experiments. (Upper left) Dose–response curves; each column represents the mean of eight wells ± SD; one-way ANOVA: **, *p* < 0.01; ***, *p* < 0.001; ****, *p* < 0.0001; (upper right) calculated IC_50_ values; (lower left) representative images of CV-stained microtiter plate rows.

To compare the antiproliferative effects of dapagliflozin and the established GCS inhibitor Genz-123346, both 3T3 and Hepa 1-6 cells were treated with increasing concentrations of the drugs for 48 h. The half-maximal inhibitory concentration (IC_50_) of dapagliflozin was determined to be 68.2 µM in 3T3 cells (Figure 4A) and 45.8 µM in Hepa 1-6 cells (Figure 4C). In contrast, Genz-123346 achieved 50% growth inhibition at much lower concentrations, 15.3 µM in 3T3 cells (Figure 4B) and 7.3 µM in Hepa 1-6 cells (Figure 4D), demonstrating its greater efficacy.

### 2.4. Dapagliflozin Does Not Inhibit GSL Synthesis

**Figure 5:**
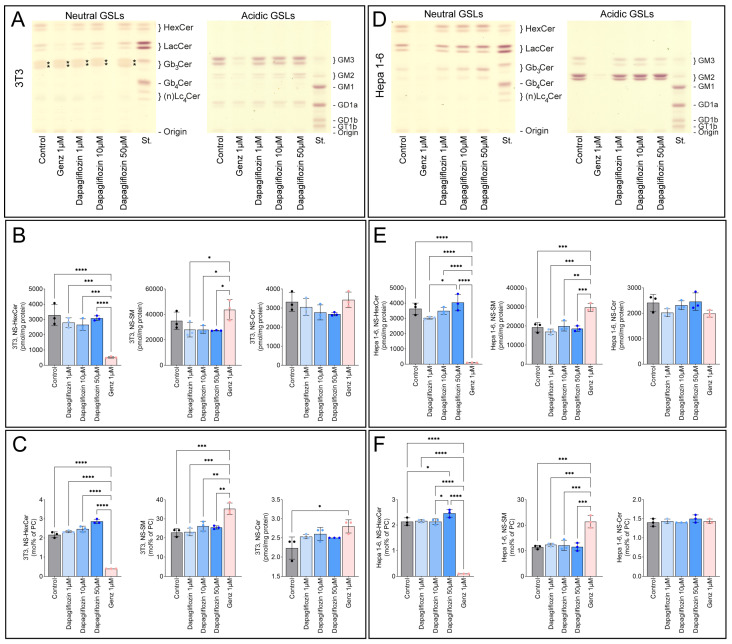
Dapagliflozin does not inhibit GSL synthesis. 3T3 cells (**A**): thin layer chromatography (TLC); (**B**,**C**): MS quantification) and Hepa 1-6 cells (**D**): TLC; (**E**,**F**): MS quantification) were treated with 1, 10, or 50 µM dapagliflozin or 1 µM Genz-123346 for six days (*n* = 3). Cells were harvested, spiked with internal standards, extracted, and analyzed by MS/MS. Hexosylceramide (HexCer/GlcCer), sphingomyelin (SM), and ceramide (Cer) were quantified relative to protein content (**B**,**E**) or internal phosphatidylcholine (PC) (mol% of PC) (**C**,**F**). Bar graphs show mean ± SD. One-way ANOVA: *, *p* ≤ 0.05; **, *p* < 0.01; ***, *p* < 0.001; ****, *p* < 0.0001. For TLC (**A**,**D**), pooled GSLs from three samples were separated into neutral and acidic (ganglioside) fractions, and material corresponding to 200 µg protein was loaded on TLC plates, developed, and stained.

As suggested by computational predictions [6], we investigated whether dapagliflozin could inhibit GSL synthesis. Hepa 1-6 cells were treated with 1 µM, 5 µM, or 10 µM dapagliflozin for four days (Figure S1A) or six days (Figure S1B), but by thin layer chromatography (TLC), no significant changes in GSL synthesis were detected. To further explore this, we increased the dapagliflozin concentration to 50 µM and included Genz-123346 as a positive control for GSL inhibition. In addition to Hepa 1-6 cells, the analysis was extended to 3T3 cells. As expected, treatment with 1 µM Genz-123346 markedly reduced GSL levels in both 3T3 cells (520 ± 55 pmol/mg protein vs. 3287 ± 703 pmol/mg in controls; Figure 5A,B) and Hepa 1-6 cells (93 ± 6.2 pmol/mg vs. 3635 ± 370 pmol/mg in controls; Figure 5D,E). In contrast, dapagliflozin had no effect on either GSL levels or sphingolipid composition at any concentration tested. Comparison of GSL expression to endogenous phosphatidylcholine (PC) in mol% (Figure 5C,F) yielded results consistent with those normalized to protein content (Figure 5B,E). While sphingomyelin (SM) levels increased in response to Genz-123346, likely as a compensatory mechanism, ceramide (Cer) levels remained largely unchanged under all experimental conditions.

### 2.5. Dapagliflozin Does Not Inhibit GSL Neo-Biosynthesis

**Figure 6:**
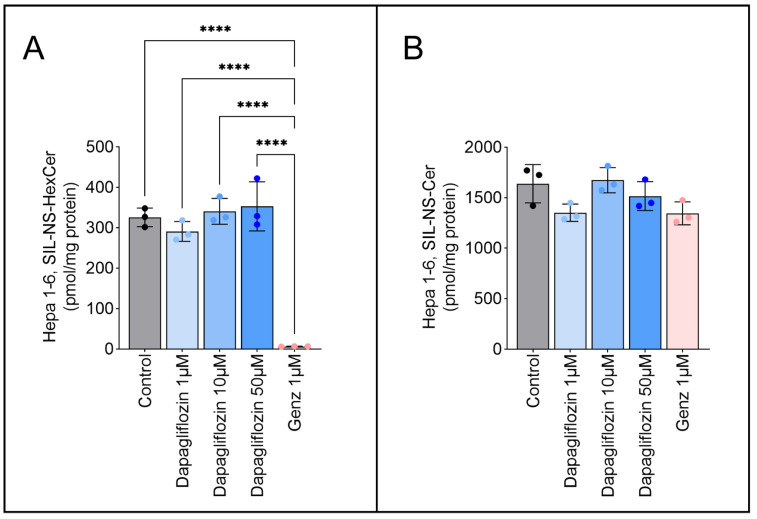
Dapagliflozin does not inhibit GSL neo-biosynthesis in Hepa 1-6 cells. Cells were pretreated with 1, 10, or 50 µM dapagliflozin or 1 µM Genz-123346 for six days. Stable isotope GSL labeling (SIL) was performed with 500 µM ^13^C_3_, ^15^N-serine for 2 h in 1% serum-containing medium (*n* = 3). Cells were harvested, spiked with internal standards, extracted, and analyzed by MS/MS. HexCer (**A**) and Cer (**B**) were quantified relative to protein content. Bar graphs show mean ± SD. One-way ANOVA: ****, *p* < 0.0001.

To determine whether dapagliflozin suppresses GSL neo-biosynthesis, Hepa 1-6 cells were treated for six days with 1 µM Genz-123346 or with 1 µM, 10 µM, or 50 µM dapagliflozin, followed by a 2 h pulse with stable isotope-labeled ^13^C_3_, ^15^N-serine (500 µM). Mass spectrometry (MRM-based MS/MS) was used to track de novo sphingolipid synthesis. Genz-123346 significantly reduced neo-synthesized GSLs to 6.6 ± 0.5 pmol/mg protein compared to 326 ± 23.1 pmol/mg in controls (Figure 6A). Dapagliflozin, however, had no measurable effect on GSL neo-biosynthesis at any concentration. Ceramide synthesis remained unaffected across all conditions (Figure 6B).

## 3. Discussion

Potent inhibition of GSL synthesis by targeting GCS, the rate-limiting enzyme in this pathway, has broad therapeutic potential. Deficiencies in glucocerebrosidase or α-galactosidase A result in the accumulation of GlcCer (Gaucher disease) or globosides (Fabry disease), respectively, leading to severe clinical manifestations. One therapeutic strategy to prevent GSL accumulation is the inhibition of GSL biosynthesis with selective GCS inhibitors [17].

Beyond lysosomal storage disorders, GSLs have emerged as promising targets in oncology. We recently demonstrated that genetic GSL depletion or pharmacological inhibition of GCS using Genz-123346 reduced GSL levels and suppressed tumor growth in murine models of chemically induced hepatocellular and colorectal cancers [18,19]. GCS inhibition has also been shown to mitigate drug resistance in cancer therapy [20,21]. However, GCS-targeting therapies are often associated with adverse effects. Miglustat, an imino sugar used in Gaucher disease, is frequently discontinued due to gastrointestinal symptoms and tremor [22]. Other GCS inhibitors, such as Genz-123346, eliglustat (Genz-112638), and PDMP, share structural features of cationic amphiphilic drugs, which can cause lysosomal lipid storage at higher concentrations. This occurs when these compounds become trapped as cations within the acidic lysosomal milieu, thereby interfering with intralysosomal degradation processes and leading to the accumulation of phospholipids and sphingolipids, including sphingomyelin, ceramides, and GSLs [19,23,24].

The search for alternative GCS inhibitors remains ongoing. Notable recent developments include T-036, tested in mice [25], and AL01211, evaluated in healthy volunteers [26]. In parallel, drug repurposing strategies aim to shorten the time required for clinical translation. Most recently, dapagliflozin was identified through computational screening as the most promising candidate in a test series for binding to the catalytic domain of GCS, thereby potentially inhibiting GSL synthesis [6]. However, this study lacked direct evidence that dapagliflozin can actually inhibit GSL synthesis. We investigated now the capacity of dapagliflozin as an inhibitor of GSL synthesis and its effectiveness as a potential anti-cancer agent in comparison with Genz-123346. Since GCS is ubiquitously expressed and highly conserved in mammalian cells, we chose murine 3T3 fibroblasts and Hepa1-6 hepatocellular carcinoma cells at random to assess the potential of dapagliflozin. We treated both cell lines with varying concentrations of dapagliflozin. Cell cycle arrest occurred only at 50 µM, whereas Genz-123346 induced measurable effects at concentrations as low as 1 µM, with pronounced effects at 5 µM [24]. Increased apoptosis upon dapagliflozin treatment was not observed, which is consistent with literature reports indicating that it rather attenuates apoptosis [27,28,29]. In the clinical treatment of type 2 diabetes mellitus, dapagliflozin is typically administered at 10 mg/day, achieving steady-state serum concentrations of ~68.6 ng/mL (~0.17 µM) after one week of administration [30]. Our data indicate that its antiproliferative effects occur only at much higher concentrations (IC_50_: 68.2 µM for 3T3 cells, 45.8 µM for Hepa1-6 cells) as compared to Genz-123346. Therefore, clinical relevance for cancer therapy seems to be limited but not excluded as SGLT2 inhibitors have been discussed as potential anti-cancer agents [31]. Dapagliflozin may also accumulate in specific organ tissues to a greater extent than in plasma, as observed in mice and minipigs [32,33,34]. We cannot rule out the possibility that, in addition to SGLT2, other membrane receptors or intracellular metabolic pathways are affected, which could help explain the observed reduction in proliferation. To the best of our knowledge, there are no reports in the literature describing serum concentrations of dapagliflozin ≥ 50 µM in humans. Moreover, it appears unlikely that such concentrations could be achieved in patients without causing pronounced side effects during long-term administration.

Moreover, dapagliflozin had no detectable effect on GSL de novo synthesis in either cell type. Even at 50 µM, GSL levels were indistinguishable from untreated controls, regardless of treatment duration. In contrast, 1 µM Genz-123346 markedly reduced GSL levels. The use of modern computational screening methods as valuable tools for drug repurposing and novel therapeutic applications is a rapidly evolving field [35]. However, depending on the analysis method applied, not all candidate hits can typically be confirmed through experimental validation [35,36]. Therefore, before a substance can be considered for clinical studies, its computational predicted functionality should first be validated in vitro.

In conclusion, dapagliflozin induces cell cycle arrest only at supra-therapeutic concentrations and does not reduce glucosylceramide levels. It therefore appears unsuitable both as an anti-cancer agent and as a therapeutic option for GSL storage disorders.

## 4. Materials and Methods

### 4.1. Chemicals

The GCS inhibitor Genz-123346 (N-[(1R,2R)-1-(2,3-dihydro-1,4-benzodioxin-6-yl)-1-hydroxy-3-pyrrolidin-1-ylpropan-2-yl]nonanamide) was obtained from Chess (Mannheim, Germany). Dapagliflozin was purchased from Thermo Scientific (Schwerte, Germany; #464620010).

### 4.2. Cell Culture

Murine 3T3 fibroblasts (generously provided by H. Wiegandt) and Hepa 1-6 hepatocellular carcinoma epithelial cells (generously provided by U. Klingmüller, this institution) were cultured in high-glucose DMEM (Sigma, Munich, Germany #D5796), supplemented with 1% penicillin/streptomycin (Thermo Fisher Scientific (Invitrogen), Waltham, MA, USA #15140-122), 1% HEPES (Thermo Fisher Scientific (Invitrogen), Waltham, MA, USA, #15630-056), and 10% FCS (Invitrogen, #A5256801). The medium was changed two to three times per week, and cells were passaged upon reaching ~90% confluency.

### 4.3. BrdU Incorporation Assay

3T3 and Hepa 1-6 cells were seeded in 10 cm dishes and treated with either 1 µM, 10 µM, or 50 µM dapagliflozin, for 4 days. Cells were harvested by trypsinization, counted, and 1.5 × 10^5^ cells (or 3 × 10^5^ cells for 50 µM dapagliflozin treatment) were seeded in triplicate into 6-well plates. Treatment continued for an additional 2 days. BrdU (Sigma, #B5002) was added to a final concentration of 10 µM, and cells were incubated for 1 h. Following BrdU incorporation, cells were washed with PBS, trypsinized, and centrifuged (400× *g*, 5 min). Cells were then resuspended in 125 µL ice-cold PBS and fixed by dropwise addition of 350 µL ice-cold 100% ethanol with gentle vortexing. Fixation continued for 3 h on ice with intermittent mixing every 30 min. Cells were then washed with PBS, centrifuged, and resuspended in 0.5 mL of 2 M HCl/0.5% Triton X-100 for 30 min at room temperature, vortexing briefly every 10 min to prevent clumping. After centrifugation and supernatant removal, cells were neutralized in 1 mL of 0.1 M Na_2_B_4_O_7_ (pH 8.5) for ~5 min on ice, then washed with 2 mL PBS containing 1% BSA. After centrifugation, cells were stained with 100 µL of 1:20 diluted APC-conjugated anti-BrdU antibody (Biolegend, San Diego, CA, USA #364114) in 1% BSA/0.5% Triton X-100 in PBS for 30 min at room temperature in the dark. Cells were washed with 1 ml of 1% BSA/0.5% Triton X-100 in PBS, then resuspended in 200 µL of 38 mM sodium citrate, 54 µM propidium iodide, and 24 µg/mL RNase A (AppliChem, Darmstadt, Germany #A38320250) and incubated for 30 min at 37 °C in the dark. FACS analysis was performed using a BD FACS Canto II system and Diva software (v9.0). Data were analyzed using FlowJo software (v3.0). Two independent experiments were performed; *n* = 3 biological replicates for each condition and cell line.

### 4.4. Cell Viability Assay

Cells were treated with 1 µM, 10 µM, or 50 µM dapagliflozin for 6 days as described. After harvesting by trypsinization and washing with 2 mL of 1% BSA in PBS, 1 × 10^5^ cells were resuspended in 100 µL of annexin binding buffer (FITC Annexin V Apoptosis Detection Kit with 7-AAD, Biolegend San Diego, CA, USA #640922), stained according to the manufacturer’s instructions, and analyzed by flow cytometry [24]. Two independent experiments were performed; *n* = 3 biological replicates for each condition and cell line.

### 4.5. Crystal Violet Proliferation Assay

3T3 and Hepa 1-6 cells were seeded in 96-well plates at densities of 4 × 10^3^ and 2 × 10^3^ cells per well, respectively, in 100 µL of medium. After overnight adherence, cells were treated with various concentrations of dapagliflozin (0.5 to 200 µM) or Genz-123346 (0.5 to 40 µM; and up to 80 µM for 3T3 cells), diluted 1:1000 in medium from ethanol stock solutions. Control cells received vehicle only (1:1000 ethanol in medium). Cells were incubated for 48 h at 37 °C and 5% CO_2_. After treatment, cells were fixed with 4% paraformaldehyde for 10 min, washed with PBS, and air-dried for ~30 min. Crystal violet solution (0.1% in 20% ethanol) was added (100 µL/well) and incubated for 30 min. Plates were washed four times with ddH_2_O and dried overnight. Stained dye was solubilized in 100 µL of 10% acetic acid by shaking at 300 rpm for 30 min, and absorbance was measured at 585 nm using a Synergy H1 microplate reader (BioTek Instruments GmbH, Bad Friedrichshall, Germany). Two to three independent experiments were performed; *n* = 3 biological replicates, respectively, for each condition and cell line.

### 4.6. GSL Analysis

In order to determine the appropriate concentrations, we first pretested dapagliflozin using increasing doses. At 100 µM dapagliflozin, cell proliferation was almost completely inhibited after four days of exposure, leaving too few cells for GSL analysis. Therefore, we selected 50 µM as the highest concentration for GSL analysis. 3T3 and Hepa 1-6 cells were treated with 1 µM, 10 µM, and 50 µM dapagliflozin or 1 µM Genz-123346. On day 4, cells were split and further cultured in the presence of the respective inhibitors for an additional 2 days as described [24]. A total of 4 × 10^6^ to 6 × 10^6^ cells were washed twice with PBS, harvested using a cell scraper, (Sartorius, Göttingen, Germany) centrifuged at 400× *g*, and the supernatant discarded. The resulting cell pellets were resuspended in 50 µL ddH_2_O. Subsequently, 0.5 mL methanol was added dropwise under gentle vortexing, followed by the addition of 0.5 mL chloroform. The samples were placed in a water bath sonicator at 37 °C for 15 min with intermittent ultrasound pulses. Following centrifugation at 1800× *g*, the supernatants were collected. The extraction of the remaining pellets was repeated twice: first with 1 mL of a chloroform/methanol/ddH_2_O mixture (10:10:1, *v*/*v*), and then with 1 mL of chloroform/methanol/ddH_2_O (30:60:8, *v*/*v*), as described above. All supernatants were combined, and both extracts and residual cell pellets were dried under a stream of nitrogen. Total protein content of the dried cell pellets was quantified using a BCA assay [24]. Crude extracts equivalent to 0.1 mg of protein were analyzed for sphingolipid content using internal standards (Cer(d18:1/14:0), Cer(d18:1/19:0), Cer(d18:1/25:0), Cer(d18:1/31:0), GlcCer(d18:1/14:0), GlcCer(d18:1/19:0), GlcCer(d18:1/25:0), GalCer(d18:1/31:0), SM(d18:1/12:0), SM(d18:1/17:0), SM(d18:1/31:0), PC(12:0/12:0), PC(14:0/14:0), PC(22:0/22:0, and PC(24:0/24:0)). Separation was performed using an Acquity I-Class UPLC system (Waters, Milford, MA, USA), coupled with a triple-quadrupole tandem mass spectrometer (Xevo TQ-S, Waters, Milford, MA, USA) to detect lipids in positive electrospray ionization and multiple reaction monitoring (MRM) mode (Supporting Tables S1 and S2). Lipids were separated on a Waters CSH C18 column (length 100 mm, diameter 2.1 mm, particle size 1.7 µm) using a gradient (Supporting Table S2) starting with 57% solvent A (50% methanol, 50% water) and 43% solvent B (99% 2-propanol, 1% methanol), both solvents containing 10 mM ammonium formiate, 0.1% formic acid and 5 mM sodium citrate as published previously [37].

For GSL analysis via TLC, crude lipid extracts were treated with 1 mL of 0.1 M KOH in methanol at 37 °C for 4 h, with occasional sonication, to eliminate phospholipids and triglycerides. Samples were neutralized with 6 µL glacial acetic acid and dried under a nitrogen stream. Salts were removed by reversed-phase column chromatography (RP18). Glass columns (2 mL) were packed with 200 µL RP18 silica gel (Chromabond #730613, Macherey-Nagel, Düren, Germany), and preconditioned sequentially with 2 mL each of methanol, ddH_2_O, and 0.1 M aqueous potassium acetate. Samples were dissolved in 1 mL ddH_2_O and applied to the columns. After two washes with 1 mL of 0.1 M potassium acetate, columns were rinsed with 3 mL ddH_2_O. Sphingolipids were eluted with 3 mL methanol. GSLs were further separated into neutral and acidic (sialic acid-containing) fractions using ion exchange chromatography. Glass columns were packed with 200 µL DEAE-Sephadex A25 (GE Healthcare, Munich, Germany) and preconditioned with 2 mL methanol. Desalted lipid samples from RP-18 columns, were directly applied to the preconditioned Sephadex-A25 columns neutral GSLs collected. Reagent tubes were rinsed twice with 1 mL methanol, which then was also applied to the columns. Columns were washed with 2 mL methanol, 2 mL chloroform/methanol/ddH_2_O (30:60:8, *v*/*v*), and 1 mL methanol to elute neutral GSLs completely. Acidic GSLs were subsequently eluted with 3 mL of 0.5 M potassium acetate in methanol and dried under nitrogen. Acidic GSLs were dissolved in 8 mL ddH_2_O and desalted again via preconditioned RP18 columns as described above. For TLC analysis, plates were loaded with sample amounts corresponding to 0.2 mg of protein. Neutral GSLs were developed using a solvent system of chloroform/methanol/ddH_2_O (62.5:30:6, *v*/*v*), and acidic GSLs using chloroform/methanol/0.2% aqueous CaCl_2_ (45:45:8, *v*/*v*). Plates were sprayed with 0.2% orcinol in 10% sulfuric acid using a CAMAG Derivatizer (CAMAG, Berlin, Germany), and GSLs were visualized by heating at 120 °C for approximately 10 min. Two independent experiments were performed; *n* = 3 biological replicates for each condition and cell line.

### 4.7. Pulse Experiment for GSL Neobiosynthesis

Hepa 1-6 cells were seeded at densities of 2 × 10^5^ (or 8 × 10^5^ for 50 µM dapagliflozin) per 10 cm dish. After 4 days of treatment with 1 µM Genz-123346 or 1/10/50 µM dapagliflozin, cells were split into triplicates (1 × 10^6^ or 2 × 10^6^ cells) and treated for another 2 days. Cells were starved for 1 h in GlutaMAX medium (Invitrogen #41090-028) supplemented with 1% FCS and 1% penicillin/streptomycin ± inhibitors, then pulsed for 2 h with 500 µM ^13^C_3,_^15^N-serine in the same medium. After the pulse, cells were washed twice with PBS, harvested in 1 mL PBS, scraped into tubes, centrifuged (400× *g*), and the pellets prepared for sphingolipid extraction as described above; *n* = 3 for each condition.

### 4.8. Statistics

One-way ANOVA test was used to compare the means of three or more independent groups in order to determine whether a metric dependent variable differed significantly between all groups. Significances were *, *p* ≤ 0.05; **, *p* < 0.01; ***, *p* < 0.001; ****, *p* < 0.0001.

## Data Availability

All data are available within the main body of the manuscript or in the Supporting Information.

## Supplementary Materials

The following supporting information can be downloaded at: https://www.mdpi.com/article/10.3390/ijms26199811/s1.

## Abbreviations

The following abbreviations are used in this manuscript:

SGLT2	Sodium-glucose cotransporter 2
GCS	Glucosylceramide synthase
GSL	Glycosphingolipid
GlcCer	Glucosylceramide
ERT	Enzyme replacement therapy
SRT	Substrate reduction therapy
BrdU	5-Bromo-2′-deoxyuridine
PI	Propidium iodide
TLC	Thin layer chromatography
MS	Mass spectrometry
(NS)-HexCer	(Non-hydroxylated and sphingosin-containing)-hexosylceramide
(NS)-SM	(Non-hydroxylated and sphingosin-containing)-sphingomyelin
(NS)-Cer	(Non-hydroxylated and sphingosin-containing)-ceramide
PC	Phosphatidylcholine
