# Supplementary material for: The SGLT2 Inhibitor Dapagliflozin Disrupts the Cell Cycle at High Concentrations Without Altering Glycosphingolipid (De Novo)Biosynthesis
Source: Int J Mol Sci. 2025 Oct 9;26(19):9811. doi: 10.3390/ijms26199811 (PMC12524616; doi:10.3390/ijms26199811)
Supplement: Supplementary file 1 [file ijms-26-09811-s001.zip › ijms-3857445-supplementary.pdf]

Supporting Figure S1

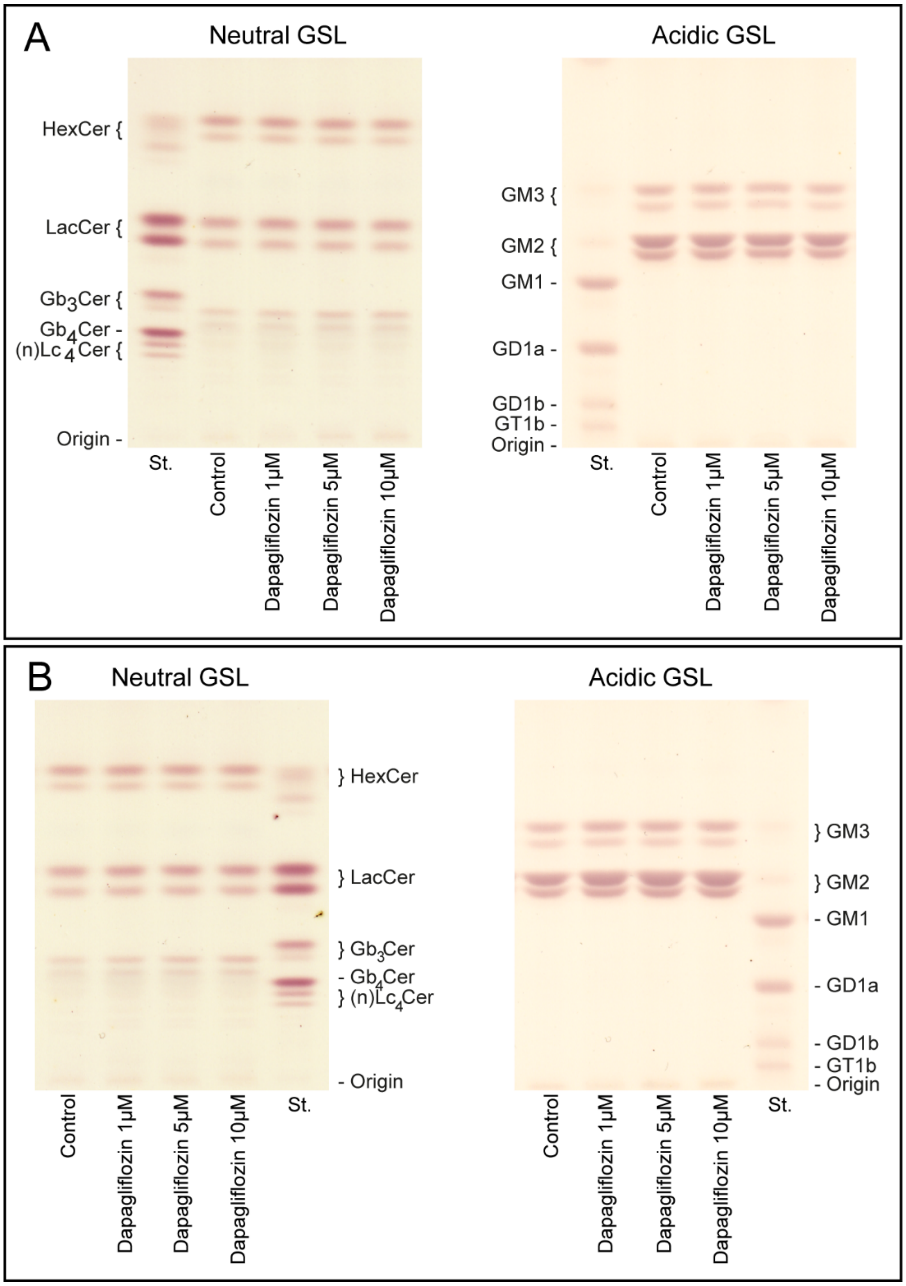

Supporting Figure S1. Dapagliflozin does not inhibit glycosphingolipid synthesis. Hepa 1-6 cells were treated with 1, 5, or 10 µM dapagliflozin for four days (**A**) or six days (**B**). Cells were harvested, extracted, and separated into neutral and acidic GSLs. Material corresponding to 200 µg protein was loaded onto thin layer chromatography (TLC) plates, developed, and visualized.

Supporting Table S1. Mass spectrometric transitions used to determine sphingolipids and phosphatidylcholine with UPLC-ESI-(QqQ)MS^2 in MRM mode

Compound (*: internal standard)		Transition		Collision energy [V]
		Precursor ion	Product ion	
NS-Ceramides (d18:1/ with 14:0*, 16:0, 18:0, 18:1, 19:0*, 20:0, 22:0, 24:0, 24:1, 25:0*, 26:0, and 31:0*)	Quantifier	$[M+H]^+$	$[M+H - (H_2O+FA)]^+$	21-23
	+ Quantifier	$[M+H - H_2O]^+$	$[M+H - (H_2O+FA)]^+$	21-23
	Qualifier	$[M+H]^+$	$[M+H - (H_2O+FA)]^+$	21-23
$^{13}C_2,^{15}N_1$-NS-Ceramides (same species as above)	Quantifier	$[^{13}C_2,^{15}N_1\text{-}M+H]^+$	$[^{13}C_2,^{15}N_1\text{-}M+H - (H_2O+FA)]^+$	21-23
	+ Quantifier	$[^{13}C_2,^{15}N_1\text{-}M+H - H_2O]^+$	$[^{13}C_2,^{15}N_1\text{-}M+H - (H_2O+FA)]^+$	21-23
	Qualifier	$[^{13}C_2,^{15}N_1\text{-}M+H]^+$	$[^{13}C_2,^{15}N_1\text{-}M+H - (H_2O+FA)]^+$	21-23
NS-HexCer (d18:1/ with 14:0*, 16:0, 18:0, 18:1, 19:0*, 20:0, 22:0, 23:0, 24:0, 24:1, 25:0*, 26:0, and 31:0*)	Quantifier	$[M+H]^+$	$[M+H - (Glc+FA)]^+$	43-47
	+ Quantifier	$[M+H - H_2O]^+$	$[M+H - (Glc+FA)]^+$	43-47
	Qualifier	$[M+H]^+$	$[M+H - (Glc+FA)]^+$	43-47
$^{13}C_2,^{15}N_1$-NS-HexCer (same species as above)	Quantifier	$[^{13}C_2,^{15}N_1\text{-}M+H]^+$	$[^{13}C_2,^{15}N_1\text{-}M+H - (Glc+FA)]^+$	43-47
	+ Quantifier	$[^{13}C_2,^{15}N_1\text{-}M+H - H_2O]^+$	$[^{13}C_2,^{15}N_1\text{-}M+H - (Glc+FA)]^+$	43-47
	Qualifier	$[^{13}C_2,^{15}N_1\text{-}M+H]^+$	$[^{13}C_2,^{15}N_1\text{-}M+H - (Glc+FA)]^+$	43-47
NS-sphingomyelin(d30:1*, d34:1, d34:2, d35:1*, d36:2, d36:1, d38:1, d40:1, d42:2, d43:1, d44:2, d44:1, and d49:1*)	Quantifier	$[M+H]^+$	$[H_2O_3PO(CH_2)_2N(CH_3)_3]^+$	35
$^{13}C_2,^{15}N_1$- NS-sphingomyelin (same species as above)	Quantifier	$[^{13}C_2,^{15}N_1\text{-}M+H]^+$	$[H_2O_3PO(CH_2)_2N(CH_3)_3]^+$	35
PC(24:0*, 28:0*, 30:1, 30:0, 32:2, 32:1, 32:0, 34:2, 34:1, 34:0, 36:5, 36:4, 36:3, 36:2, 36:1, 36:0, 38:7, 38:6, 38:5, 38:4, 38:3, 38:2, 40:8, 40:7, 40:6, 40:5, 44:0*, 48:0*)	$[M+H]^+$	$[H_2O_3PO(CH_2)_2N(CH_3)_3]^+$	35	$[M+H]^+$

* indicates internal standards.

Supporting Table S2. UPLC solvent gradient used to separate lipids

Time [min]	Flow rate	% A	% B	Type of curve
Initial	0.35	57	43	Linear (6)
0.2	0.35	57	43	Linear (6)
0.4	0.35	50	50	Linear (6)
4.0	0.35	30	70	Non-linear (7, starting slow)
10	0.35	5	95	Linear (6)
11	0.35	5	95	Linear (6)
11.5	0.35	1	99	Linear (6)
12	0.35	57	43	Linear (6)
14	0.35	57	43	Linear (6)
